# Conotoxins Targeting Neuronal Voltage-Gated Sodium Channel Subtypes: Potential Analgesics?

**DOI:** 10.3390/toxins4111236

**Published:** 2012-11-08

**Authors:** Oliver Knapp, Jeffrey R. McArthur, David J. Adams

**Affiliations:** Health Innovations Research Institute, RMIT University, Melbourne, Victoria 3083, Australia; Email: oliver.knapp@rmit.edu.au (O.K.); jeffrey.mcarthur@rmit.edu.au (J.R.M.)

**Keywords:** voltage-gated sodium channel, Na_v_1.3, Na_v_1.7, Na_v_1.8, Na_v_1.9, μ-conotoxin, μO-conotoxin, nociception, analgesic, pain

## Abstract

Voltage-gated sodium channels (VGSC) are the primary mediators of electrical signal amplification and propagation in excitable cells. VGSC subtypes are diverse, with different biophysical and pharmacological properties, and varied tissue distribution. Altered VGSC expression and/or increased VGSC activity in sensory neurons is characteristic of inflammatory and neuropathic pain states. Therefore, VGSC modulators could be used in prospective analgesic compounds. VGSCs have specific binding sites for four conotoxin families: μ-, μO-, δ- and ί-conotoxins. Various studies have identified that the binding site of these peptide toxins is restricted to well-defined areas or domains. To date, only the μ- and μO-family exhibit analgesic properties in animal pain models. This review will focus on conotoxins from the μ- and μO-families that act on neuronal VGSCs. Examples of how these conotoxins target various pharmacologically important neuronal ion channels, as well as potential problems with the development of drugs from conotoxins, will be discussed.

## 1. Introduction

Depolarization-activated sodium (Na^+^)-selective ion channels (also known as voltage-gated sodium channels, VGSCs) are large transmembrane proteins present in central and peripheral neurons, and skeletal, cardiac and smooth muscle. In response to a small depolarization of the membrane potential, VGSCs cause a large depolarization by facilitating Na^+^ entry into the cell. This signal amplification is responsible for initiating and propagating action potentials, and is the defining property of excitable cells [1]. 

VGSCs consist of a 260 kD pore-forming a-subunit, of which there are nine identified mammalian subtypes (Na_v_1.1–Na_v_1.9). Each subtype has distinct tissue distribution and biophysical properties. a-Subunits have intracellular N- and C-termini and consist of four homologous domains (DI–IV). Each domain contains six transmembrane-spanning helices (S1–S6) and can be broken down into two segments. The first is the S1–S4 segment, known as the voltage sensor, in which the positively charged S4 transmembrane segment moves in response to voltage and couples this movement to opening and closing of the pore domain. The second is the S5-P loop-S6, where ion selectivity and permeation occur. 

The structure of bacterial sodium channels Na_v_Ab and Na_v_Rh in various states have recently been determined to atomic resolution by X-ray crystallography [2,3,4]. A number of novel structural features were identified, including a short selectivity filter and ion selectivity determined by interactions with glutamate side chains [2]. Intriguingly, the crystal structures show that the channel lumen is penetrated by fatty acyl chains, via lateral fenestrations from the membrane. The fatty acyl chains extend into the central cavity, perhaps allowing small, hydrophobic channel modulators reach their binding site [2]. Studies of various toxins and drugs that interact with VGSCs have revealed clustered interaction sites on the channel. To date, nine distinct neurotoxin/drug receptor binding sites have been characterized on VGSC α-subunits (receptor sites 1–9) [5,6].

Most VGSC subtypes are blocked by nanomolar concentrations of puffer fish poison, tetrodotoxin (TTX), so are classified as TTX-sensitive (TTX-S) VGSCs. Conversely, several VGSC subtypes, such as Na_v_1.5, Na_v_1.8 and Na_v_1.9, are insensitive to nanomolar concentrations of TTX, so are classified as TTX-resistant (TTX-R) VGSCs [7]. TTX resistance has been linked to a single mutation in a residue in the channel pore. In TTX-S VGSCs, this residue is a tyrosine or phenylalanine (Y401 in Na_v_1.4), which is one residue external to the selectivity filter ring in DI (DEKA locus). However, in TTX-R channels, this residue is a cysteine or serine, which provides an unfavorable interaction with TTX, reducing the binding affinity for TTX in the pore [8,9].

VGSC α-subunits alone can form functional channels, but they may be associated with one or two auxiliary β-subunits, of which there are four known subtypes: β1 (36 kD), β2 (33 kD), β3 (25 kD) and β4 (38 kD). β-subunits comprise a single transmembrane segment, short intracellular C-terminus and large extracellular N-terminal domain harbouring two β-sheets in an immunoglobulin-like fold. Adding β-subunits to channel-forming α-subunits changes channel kinetics and voltage-dependent gating to that normally seen in native cells. Modulation of VGSC α-subunits by β-subunits is thought to mainly occur via interaction between the extracellular domains of both subunits [10]. However, two studies suggest the intracellular domains of VGSC α- and β-subunits also interact, and that this interaction may be important for modulating certain VGSC subtypes [11,12]. 

## 2. VGSC Subtypes Involved in Pain

Chronic pain can be generally classified as inflammatory or neuropathic. Inflammatory pain is caused by tissue injury or inflammation, and usually disappears as soon as the healing process ends. It includes muscle pain, headache and pain associated with cancer. Inflammatory pain can often be relieved through using non-steroidal anti-inflammatory drugs and/or morphine derivatives. Neuropathic pain may be caused by nerve injury. It is usually accompanied by a burning sensation and only slightly relieved by anaesthetics, anticonvulsants and tricyclic antidepressants [13,14]. Unlike inflammatory pain, neuropathic pain is generally characterized by allodynia, when a normally non-painful stimulus is painful. Neuropathic pain is estimated to affect millions of people worldwide [15]. It reduces the affected person’s overall health and quality of life, while generating healthcare costs several times higher than those for control groups [16,17].

The molecular basis of inflammatory and neuropathic pain appears to involve changes in ion channel activity and/or expression levels, including that in VGSCs. For example, changes in VGSC expression in sensory neurons changes neuronal excitability [18,19,20,21,22,23,24,25,26,27,28,29]. VGSC subtypes Na_v_1.3, Na_v_1.7, Na_v_1.8 and Na_v_1.9 are believed to be specifically involved in transmitting pain signals. Irregular function of these VGSCs has been shown to produce harmful or even fatal channelopathies [30]. Therefore, therapeutics designed to target one of these four VGSC isoforms must be highly selective.

Certain groups of sensory neurons are the main mediators of pain sensation. Sensory neurons transmit information from the periphery to the spinal cord. The spinal cord then transfers this information to the brainstem and forebrain. Nerve damage may cause hyperexcitability by increasing the firing frequency and/or generation of spontaneous action potentials from dorsal root ganglion (DRG) neurons [31]. 

### 2.1. Na_v_1.3

Na_v_1.3 is a TTX-S VGSC subtype normally expressed in the central nervous system (CNS). More Na_v_1.3 is usually expressed in embryogenesis than adulthood [32], but expression levels increase in peripheral DRG neurons after nerve injury and inflammation [33]. This suggests Na_v_1.3 plays a role in pain sensation [34]. Na_v_1.3 is also up-regulated in mammalian DRGs after spinal cord [35] and motor fiber injury [36]. Patients with trigeminal neuralgia have increased Na_v_1.3 expression in gingival tissue [37]. 

Na_v_1.3 has faster kinetics and recovers more quickly from inactivation than TTX-R VGSCs [38]. Therefore, it could play an important role in high-frequency firing, as seen in chronic pain states [22,39]. Reducing Na_v_1.3 expression in pain-sensing peripheral neurons has been shown to diminish hypersensitivity in DRGs and pain [33]. However, Nassar *et al.* showed that Na_v_1.3 knock-out mice still exhibit normal neuropathic pain behaviour and ectopic discharges from damaged nerves [40]. This suggests there may be a possible compensation mechanism in knock-out mice. 

### 2.2. Na_v_1.7

Na_v_1.7 is a TTX-S VGSC subtype, predominantly restricted to the peripheral nervous system (PNS), sensory and sympathetic neurons, and Schwann and neuroendocrine cells [41,42,43,44,45]. It displays rapid activation and inactivation kinetics [46]. 

Recently, Na_v_1.7 received attention as a possible therapeutic target for drugs to treat pain. Mutations in the SNC9A gene, which encodes Na_v_1.7, have been shown to produce or prevent pain. Gain-of-function mutations, which produce pain, lead to a syndrome called ‘primary erythromelalgia’, a paroxysmal extreme pain disorder [47,48]. This syndrome is characterized by severe burning pain and redness in the extremities. In contrast, loss-of-function mutations, which prevent pain, lead to a syndrome called ‘congenital insensitivity to pain’ or ‘congenital analgesia’ [49,50,51]. This divergent spectrum, from feeling no pain to feeling constant pain, makes Na_v_1.7 an interesting potential therapeutic target. Consequently, Na_v_1.7 blockers are prospective analgesic compounds. For example, a peripherally acting Na_v_1.7-specific blocker, *N*-[(*R*)-1-((*R*)-7-chloro-1-isopropyl-2-oxo-2,3,4,5-tetrahydro-1*H*-benzo[*b*]azepin-3-ylcarbamoyl)-2-(2-fluorophenyl)-ethyl]-4-fluoro-2-trifluoromethyl-benzamide (BZP), has been shown to be as effective as the analgesic drugs gabapentin and mexiletine in reversing hyperalgesia and allodynia in rat models of inflammatory and neuropathic pain, but did not impair motor function [52].

Na_v_1.7 has also been shown to be necessary for odour perception in rats, mice and humans [53]. Humans with loss-of-function mutations in the SCN9A gene couldn’t detect odours, and although Na_v_1.7-null mice neurons still produced odour-evoked action potentials, they couldn’t initiate synaptic signalling from their axon terminals at the first synapse in the olfactory system. As a result, these mice no longer displayed vital, odour-guided behaviours [54]. Na_v_1.7 has also been shown to be expressed in rat olfactory sensory axons and is present in vomeronasal axons, indicating a role for Na_v_1.7 in transmitting pheromonal cues [55]. In addition, neuroepithelial injury caused transient expression of Na_v_1.7 by dendritic cells of monocytic lineage, suggesting an emerging role for this channel in immune function [55]. 

### 2.3. Na_v_1.8

Although Na_v_1.8 is selectively expressed in sensory neurons and C-type nerve fibers involved in nociception, mediating a slow-inactivating, TTX-R Na^+^ current, its precise role in pain is unclear. Peripheral nerve injury has been shown to reduce Na_v_1.8 expression in damaged neurons, indicating that Na_v_1.8 does not contribute to neuropathic pain [56]. However, Na_v_1.8 has also been shown to redistribute to the axons of uninjured sciatic nerves after spinal nerve ligation, indicating it plays a crucial role in pain states [56]. Zimmermann *et al.* also showed that Na_v_1.8 is essential for nociception in the cold and for cold pain [57]. Changes in Na_v_1.8 gene expression are partially attributed to changes in growth factor levels and altered auxiliary β-subunit expression levels [58,59]. 

Na_v_1.8 knock-out mice display increased pain behaviour in comparison with wild-type mice, providing further evidence for a major role for this channel in pain [18,60,61]. Furthermore, discovery of the μO-conotoxin MrVIB, a potent and preferential Na_v_1.8 inhibitor, has provided evidence that blocking Na_v_1.8 can alleviate chronic pain in rats [61,62]. Nevertheless, the role of Na_v_1.8 in neuropathic pain remains a matter for debate [37,45,60,63,64,65]. 

### 2.4. Na_v_1.9

Na_v_1.9 is a TTX-R VGSC subtype whose expression is restricted to the PNS. Its amino acid sequence exhibits only around 50% homology to that of other VGSCs. Na_v_1.9 elicits extraordinarily slow persistent currents and its activation kinetics are too slow to contribute to the action potential’s upstroke. Accordingly, Na_v_1.9 probably depolarizes the resting membrane potential, lowering the threshold for initiating action potentials [66,67,68]. 

Studies using Na_v_1.9-null mice suggest this subtype plays a predominant role in inflammatory, but not neuropathic, pain [69,70]. However, the role of Na_v_1.9 in inflammatory pain is not completely clear, because Na_v_1.9 knockdown mediated by anti-sense oligodeoxynucleotides doesn’t reduce thermal hypersensitivity associated with complete Freund’s adjuvant-induced inflammatory pain [71]. It has been suggested that Na_v_1.9-mediated currents are down-regulated after nerve injury, but other studies contradict this idea [22,64]. 

## 3. Conotoxins

Conotoxins are small peptides in the venom of predatory, tropical marine cone snails from the genus *Conus.* Cone snails can be divided into three groups based on their primary prey: piscivorious (fish-hunting), vermivorous (worm-hunting) or molluscivorious (mollusc-hunting). 

Cone snails first synthesize propeptides in the secretory cells of their tubular venom duct. The precursor protein is then cleaved by proteases, generating active conotoxins that form key constituents of the venom. The cone snail injects its venom into prey using a harpoon-like, specialized radular tooth [72]. Venom is primarily used to immobilize prey, giving the cone snail sufficient time to engulf it, but can also be used for defense. Although injecting venom typically rapidly paralyzes prey, there are documented cases of venom from some fish-hunting species (e.g., *Conus geographus*) causing human fatalities [73,74]. 

All cone snail venoms are complex, with each cone snail species producing a cocktail of more than 1000 distinct peptides in the venom [75]. These peptides have a diverse range of pharmacological targets, including membrane ion channels like VGSCs. To date, four conotoxin families that target VGSCs have been identified: μ-, μO-, δ- and ί-conotoxins. Each of these families interact with VGSCs by binding to specific sites on the channel protein. 

μ-Conotoxins (Table 1) inhibit the current through open VGSCs by sterically and electrostatically blocking the ion-conducting pathway by binding to the outer vestibule of the channel [76]. Similar to μ-conotoxins, μO-conotoxins (Table 2) also inhibit VGSC currents. However, they do this by binding to a site external to the pore, which modifies channel gating and closes the channel [77]. 

Recently two new conotoxins of the Τ-superfamily (two disulfide bonds), Cal12a and Cal12b, isolated from *Conus californicus* and one of the O2-superfamily (four disulfide bonds), LtVD, from *Conus litteratus* have been identified [78,79]. These peptides have been shown to inhibit VGSCs in either rat DRG neurons (LtVD [79]) or squid stellate ganglia neurons (Cal12a, Cal12b [78]). These disulfide-bonded peptides may represent new conotoxin families that target neuronal VGSCs. 

Unlike μ- and μO-conotoxins, δ-conotoxins activate VGSCs. They do this by potentially interacting with hydrophobic surface residues of the S3/S4 linker of DIV, site 6. Domain IV is known to be critical for channel inactivation. This interaction extends channel openings, which prolongs action potentials and triggers persistent neuronal firing, ultimately delaying or inhibiting fast inactivation [73,80]. δ-Conotoxins consist of approximately 30 amino acids, contain three disulfide bonds that form an inhibitory cysteine knot, and are structurally similar to μO- and ω-conotoxins [81,82]. δ-Conotoxins from mollusc-hunting cone snails target only mollusc sodium channels and do not affect mammalian VGSCs. Very little is known about their subtype specificity [83,84,85,86,87]. 

ί-Conotoxins also activate VGSCs, but unlike δ-conotoxins, do this without significantly affecting inactivation. They may do this by enhancing the amplitude of the TTX-S Na^+^ current in DRG neurons, or shifting the voltage dependence of activation to more hyperpolarized potentials. Both mechanisms do not affect the time course of inactivation [88,89]. 

To date, the δ- and ί-conotoxins have not exhibited any analgesic activity and only limited data on their mode of action or subtype specificity is available. Therefore, in this review we will focus on μ- and μO-conotoxins.

### 3.1. μ-Conotoxins

μ-Conotoxins are highly basic, small, rigid peptides ranging from 16 to 26 amino acids long, and contain three disulfide bonds (Table 1). μ-Conotoxins bind to the pore region of VGSCs. Their selectivity for VGSC subtypes has been based predominantly on differences in the turret region (S5-P loop linker), because differences close to the pore are minimal [90]. 

**Figure 1:**
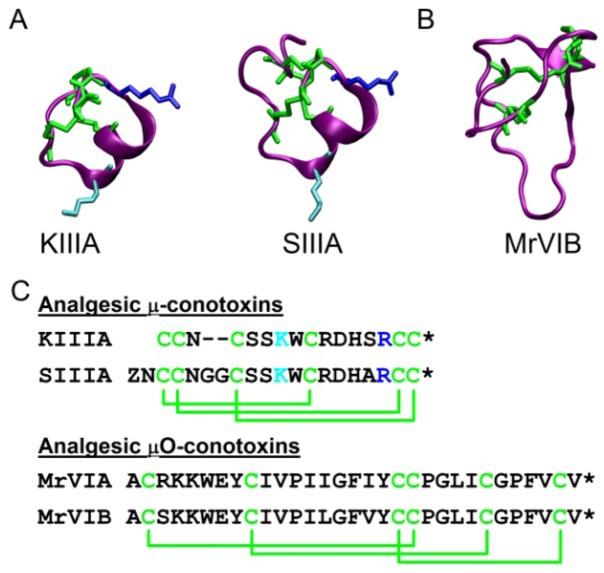
Structures and sequences of analgesic μ- and μO-conotoxins. **(A)** Solution structures of analgesic μ-conotoxins, KIIIA and SIIIA. Disulfide links (green) with critical lysine (cyan, K7 (KIIIA) and K11 (SIIIA)) and arginine (blue, R14 (KIIIA) and R18 (SIIIA)) residues. **(B)** Solution structure of analgesic μO-conotoxin MrVIB. Disulfide links are green. **(C)** Amino acid sequences of analgesic μ-conotoxins, KIIIA and SIIIA, and μO-conotoxins, MrVIA and MrVIB. * C-terminal amidation.

The first μ-conotoxin isolated and characterized was GIIIA from *C. geographus*. GIIIA was shown to be a potent and selective blocker of muscle VGSCs [91]. It inhibits the skeletal muscle VGSC, Na_v_1.4, with an IC_50_ in the low nanomolar range [92,93]. GIIIA’s ability to selectively target a single VGSC subtype suggests there may be other μ-conotoxins that could selectively target different VGSC subtypes, particularly those involved in pain signalling. Since the discovery of GIIIA, numerous μ-conotoxins with new sequences have been identified through molecular cloning techniques. Many of their structures have been solved using NMR spectroscopy (Figure 1) [94,95,96,97,98]. 

The first μ-conotoxin shown to inhibit a neuronal VGSC was PIIIA. Despite having modest affinity for the neuronal channel Na_v_1.2 (~500 nM), PIIIA primarily blocks VGSCs in mammalian skeletal muscle [99]. 

Another μ-conotoxin, KIIIA, was recently shown to preferentially bind to the neuronal channel Na_v_1.2 over the skeletal muscle channel Na_v_1.4. KIIIA also has a nanomolar affinity for Na_v_1.7, a VGSC involved in propagating pain signals [100]. Being only 16 amino acids long, KIIIA is considerably shorter than previously examined μ-conotoxins. The short length is due to fewer residues within the N-terminal half of the peptide, suggesting C-terminal residues are more critical for toxin binding than N-terminal residues. 

KIIIA and another small μ-conotoxin, SIIIA, have been shown to have analgesic properties in mouse inflammatory pain assays [101,102,103]. These μ-conotoxins have a framework that could be used to develop new analgesic drugs. Their small size, high affinity and unique VGSC channel selectivity make them strong prospective therapeutic agents. 

**Table 1:** Neuronal voltage-gated sodium channels (VGSC) subtype selectivity and potency of μ-conotoxins.

μ-Conotoxins	*Conus* species	Number of residues	VGSC subtypes (IC_50_)	References
KIIIA	*C. kinoshitai*	16	Na_v_1.3 (8 μM),	[100,101]
			Na_v_1.7 (100–290 nM)	
SIIIA*/B	*C. striatus*	20/20	Na_v_1.3 (11 μM (SIIIA)),	[103,104,105]
			Na_v_1.7 (65 μM (SIIIA)),	
			Na_v_1.8 (insensitive to 10 μM)	
PIIIA	*C. purpurascens*	22	Na_v_1.3 (3.2 μM),	[96,106,107]
			Na_v_1.7 (3.1–6.2 μM)	
GIIIA/B/C	*C. geographus*	22/22/22	N.D.	
CnIIIA/B/C	*C. consors*	22/25/22	Na_v_1.3 (11 μM (CnIIIA)),	[98,107,108]
			Na_v_1.7 (489 nM (CnIIIC))	
			Na_v_1.8 (insensitive to 1 μM (CnIIIC))	
CIIIA	*C. catus*	22	N.D.	
SmIIIA	*C. stercusmuscarum*	22	Na_v_1.3 (40 nM),	[97]
			Na_v_1.7 (1.3 μM)	
MIIIA	*C. magus*	22	Na_v_1.3 (7.7 nM),	[107]
			Na_v_1.7 (97 μM)	
SxIIIA/B	*C. striolatus*	22/23	N.D.	
BuIIIA/B/C	*C. bullatus*	23/24/26	Na_v_1.3 (350 nM (BuIIIA); 200 nM (BuIIIB))	[107]
TIIIA	*C. tulipa*	22	Na_v_1.3 (50 nM),	[107]
			Na_v_1.7 (insensitive to 3 μM)	
			Na_v_1.8 (insensitive to 3 μM)	

N.D., not determined. *commercial name PEG-SIIIA; entered pre-clinical trial for inflammatory pain (i.v.) [109].

μ-Conotoxins bind to the outer vestibule to inhibit current through the channel. To understand which of the four channel domains are critical to binding, chimeras between rat Na_v_1.4 (rNa_v_1.4) and human Na_v_1.5 (hNa_v_1.5) or human Na_v_1.8 (hNa_v_1.8) have been constructed and examined [104,108]. These studies showed that SIIIA and CnIIIC are potent blockers of the VGSC subtypes Na_v_1.2 and Na_v_1.4, but inactive at Na_v_1.8 and Na_v_1.5. The studies further revealed that determinants for the toxin’s inability to block Na_v_1.8 were located across all domains of the channel, whereas those for Na_v_1.5 were restricted to DI and DII. 

There are few differences among the VGSC isoforms in the pore region near the inner and outer ring charge groups (important for ion selectivity and permeation) of the outer vestibule in each of their four channel domains. However, a single amino acid residue change in this region has been shown to be critical for classical pore blockers saxitoxin and TTX [7]. This residue is located in DI, one residue outside the selectivity filter aspartate. It is either a tyrosine or phenylalanine in TTX-S channels, and a serine or cysteine in TTX-R sodium channels. 

This variation among sodium channel isoforms also affects μ-conotoxin binding properties, such as affinity, but this change is modest [100,110]. Nevertheless, it modulates the actions of various μ-conotoxins analogues that incompletely block single channel currents [106]. Potential derivatives that do not completely block single channel currents could fine tune hyperexcitability. Derivatives of GIIIA and PIIIA as well as KIIIA and its derivatives, which have been examined at the single channel level, cannot completely block single channel currents [93,101,110] and could be used in this way. A drug that modulates single-channel currents would be an effective treatment to reduce hyperexcitability without eliminating Na^+^ channel currents completely.

An additional variation in the sequence in this VGSC region is attractive to examine because it may identify potential human Na_v_1.7 (hNa_v_1.7) blockers. Human Na_v_1.7 is the only VGSC subtype, from any species, where the DIII outer ring charge (typically an aspartate) has been replaced by a neutral isoleucine. μ-Conotoxin binding affinity is critically influenced by residues in the outer ring, especially those of DI and DII [111,112,113]. Recent studies using KIIIA showed that charges interacting with this outer ring charge, when neutralized, affected the derivative’s affinity for hNa_v_1.7 much less than its affinity for Na_v_1.2 and Na_v_1.4 [100]. The missing outer ring charge of hNa_v_1.7 is believed to be the best site to engineer toxin specificity towards hNa_v_1.7. For example, in KIIIA, the derivative H12Q, which, based on molecular dynamics docking simulations, should interact with the DIII outer ring charge, increased toxin selectivity for hNa_v_1.7 over Na_v_1.2 and Na_v_1.4. The R14A derivative increased the toxin’s selectivity for hNa_v_1.7 to 10-fold more than that for Na_v_1.2 and Na_v_1.4 [100]. This is the first example of an engineered μ-conotoxin that is selective for hNa_v_1.7. This μ-conotoxin interaction site is a potential target for designing hNa_v_1.7-specific blockers, which could increase selectivity towards Na_v_1.7 and decrease the risk of possible side effects.

### 3.2. μ-Conotoxin Engineering

Recent studies using new peptide synthesis techniques are shedding light on how to create stable and selective sodium channel modulators, and identifying new drug leads. Peptides as drug leads have a few drawbacks, such as unstable disulfide bonds, protease degradation and inefficient transport of the peptide to its intended site of action. To address these concerns, researchers have been trying to improve the native μ-conotoxin by creating more stable peptide bridges, using truncated peptides and/or peptide mimetics. They have also used μ-conotoxins as scaffolds to enhance the peptide’s potential as a drug. 

μ-Conotoxins have three disulfide bonds, which can be oxidized and reduced, and refolded to inactive conformations. This was examined in detail for PIIIA, where three PIIIA isomers were created with different disulfide connections [114]. Despite differences in connectivity, the three derivatives all had sub-micromolar affinity for Na_v_1.4. This suggests that, despite the toxin preferring to fold to a single conformation, other conformations could also be active. It will be of interest to examine these different PIIIA isomers to determine if they exhibit different selectivity to that of the native toxin. 

To make the peptide structure stable, different non-disulfide bonds can also be introduced. For example, SIIIA with diselenide bridges instead of disulfide bridges was recently synthesized [115]. Diselenide bonds are considerably more stable than disulfide bonds, making the μ-conotoxin itself more stable [116].

With many new μ-conotoxin sequences being discovered, there seems to be greater divergence in the N-terminus half of the toxin sequence. For example, KIIIA seems to be missing many more residues in the N-terminus than other μ-conotoxins. It may be interesting to examine the effects of smaller μ-conopeptides by removing N-terminal residues. Several groups recently examined short peptides based on KIIIA and SIIIA, with varying success. Norton and colleagues used lactam bridges to stabilize the α-helical segment of μ-conotoxins [117,118]. This segment has most of the critical residues for toxin binding and might be used as a template on which to base drug design. KIIIA and BuIIIC were used to design ‘mini peptides’ ranging from 12 to 16 amino acids, to try to understand the pharmacophore of μ-conotoxins [119,120]. 

In contrast with these two studies, adding residues to the N-terminus of SIIIA, SIIIB and TIIIA has been shown to change their selectivity preference from rat muscle channels to rat neuronal VGSCs [121]. This suggests that N-terminal residues also contribute to the toxins’ affinity and selectivity.

As peptides are degraded or truncated by proteases, non-peptide backbones have also been examined to try to increase drug bioavailability. KIIIA and SIIIA structure has undergone non-peptide modification to help design new drug leads. For example, Bulaj and colleagues examined the effects of removing a single disulfide bond to see if it is feasible to use non-peptide backbone spacers and disulfide bond deletion to increase bioavailability without decreasing affinity or change selectivity. They also used amino-3-oxapentanoic or 6-aminohexanoic acids to replace non-essential amino acids in the toxin to further minimize the protein component of the toxin [103,122]. Resulting analogues still inhibited VGSCs and produced analgesic effects. 

μ-Conotoxins as potential analgesics are still in the preclinical stage. We are learning how these unique toxins can block specific VGSC subtypes, and how we can modify them without changing their selectivity and high affinity for VGSCs. As we further examine these toxins and isolate new toxins that can block specific VGSC subtypes, we will be able to design new selective and clinically relevant drugs to block VGSC subtypes involved in pain signalling.

### 3.3. μO-Conotoxins

μO-Conotoxins are hydrophobic peptides belonging to the O-superfamily of conotoxins. This group of toxins was first isolated from *Conus marmoreus* [82]. To date, five μO-conotoxins have been discovered (Table 2). They range from 28 to 32 amino acids in length, with each toxin containing three intramolecular disulfide bonds. Two of these μO-conotoxins, MrVIA and MrVIB, have high sequence homology. Only two residues differ between them. MrVIA and MrVIB are of interest because they block TTX-R Na^+^ currents in mammalian DRG neurons (which are mainly mediated by Na_v_1.8) 10-fold more than TTX-S Na^+^ currents [62,123,124]. However, despite the selectivity of MrVIA and MrVIB for the TTX-R subtype Na_v_1.8 over TTX-S subtypes, and the resulting analgesic activity in a variety of animal models of pain [62,123,124,125], little is known of the structure–activity relationships that define μO-conotoxin subtype specificity. 

MrVIA inhibits VGSCs and voltage-gated calcium channels (VGCC) in *Aplysia californica* and *Lymnaea stagnalis* molluscan neurons [82], and amphibian VGSCs [124]. It also inhibits TTX-S Na^+^ currents in rat hippocampal neurons and heterologously expressed rNa_v_1.2 in *Xenopus* oocytes with IC_50_ values of 200 nM [126]. MrVIA blocks TTX-R VGSCs from rDRG neurons with an IC_50_ of 82.8 nM [125]. Consistent with μO-conotoxins acting as gating modifiers, MrVIA inhibition of Na^+^ current appears to be voltage-dependent, with a reduced affinity for the channel after depolarizing voltage steps [77,127]. 

MrVIB more selectively inhibits TTX-R than TTX-S neuronal VGSCs, and is also selective between different TTX-R VGSC subtypes. It is 100-fold more selective for Na_v_1.8 than Na_v_1.9 in DRGs [62]. Its selectivity for hNa_v_1.8 over other VGSC subtypes was confirmed using *Xenopus* oocytes expressing a range of different VGSC subtypes [62]. This is particularly interesting, since only a few Na_v_1.8-selective modulators have been described to date. Zorn *et al.* also showed that MrVIB is more selective for Na_v_1.4 than Na_v_1.2 expressed in HEK 293 cells [128]. 

MrVIB has exhibited analgesic activity in animal pain models and decreased mechanical and thermal pain sensation [62,125]. Intrathecally applied MrVIB was 30 times more effective than lidocaine on allodynia and hyperalgesia and had only small motor system side effects [62]. The 3D structure was determined using NMR data (Figure 1) [125].

There is considerable interest in finding out how μO-conotoxins bind to VGSCs, since they are modestly selective inhibitors of TTX-R VGSC currents in rat DRG neurons [125] and have potential as analgesic drugs [62,123,124]. Analysis of the MrVIA binding site in rNa_v_1.4 channels and competition experiments with the scorpion toxin, β-toxin Ts1, identified that the C-terminal pore loop of DIII is necessary for MrVIA to bind to Na_v_1.4 [128]. In contrast, a later study using site-directed Na_v_1.4 mutagenesis, revealed that DII’s voltage sensor is the main MrVIA binding site. The same study concluded that MrVIA interaction with DIII of Na_v_1.4 appeared to play a lesser role [77]. Neither of these two studies examined the binding site and mechanism of action of μO-conotoxins on Na_v_1.8. 

**Figure 2:**
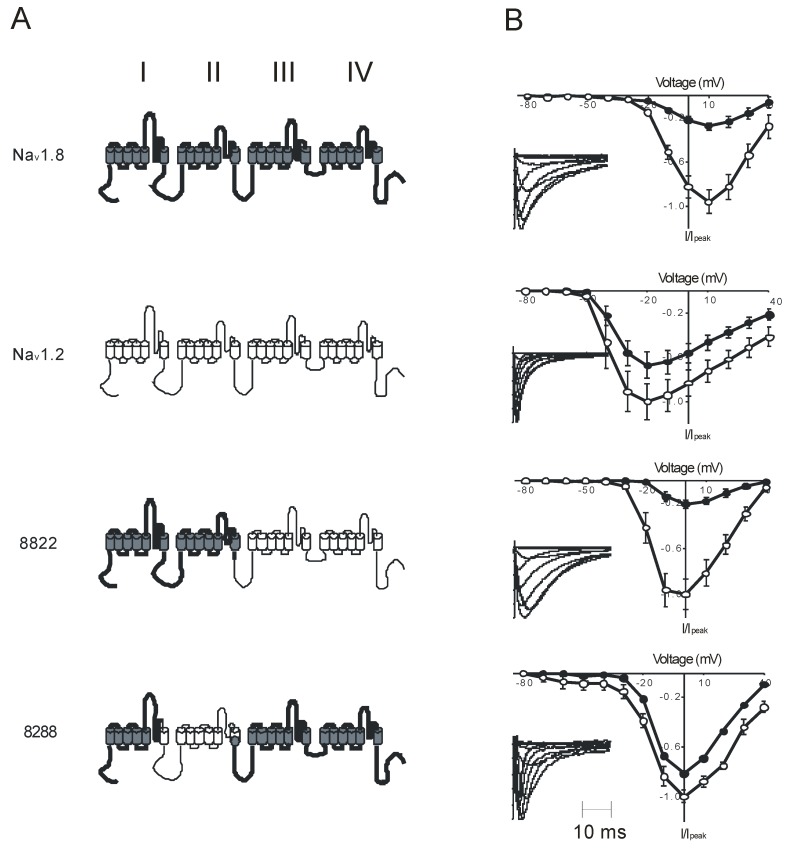
Effect of μO-conotoxin MrVIB on Na^+^ current amplitude of Na_v_1.2, Na_v_1.8 and their chimeras. **(A)** Schematic diagram of parent VGSC a-subunits rNa_v_1.2 (open), hNa_v_1.8 (filled) and chimeras 8822 and 8288. Roman numerals denote individual domains of the α-subunit, **(B)** Current–voltage relationship of rNa_v_1.2, hNa_v_1.8 and chimeras 8288 and 8822 in the absence (open symbols) and presence (closed symbols) of 1 μM MrVIB. Normalized peak currents (I/I_0_) were plotted as a function of membrane potential. Insets: normalized depolarization-activated Na^+^ currents in *Xenopus* oocytes expressing rNa_v_1.2, hNa_v_1.8 and the chimeras. Oocytes were held at −80 mV and depolarized to potentials ranging from −80 to +40 mV in 10 mV increments (adapted from Knapp *et al.* [129]).

Na_v_1.8 is only 50% identical to Na_v_1.2, a major VGSC of the central nervous system, and its affinity for MrVIB is higher than that of Na_v_1.2 [62]. To address the functional and pharmacological significance of these differences, Knapp *et al.* used a domain swapping strategy between rNa_v_1.2 and hNa_v_1.8 [129]. Heterologous expression of Na_v_1.2/Na_v_1.8 chimeras in *Xenopus* oocytes and analysis of their inhibition by MrVIB revealed that the region between segment S6 of DI and the external loop of DII in Na_v_1.8 is the main determinant for the μO-conotoxin family’s high affinity for Na_v_1.8 (Figure 2) [129]. Comparing this data with those of Leipold *et al.* supports the likelihood that MrVIB inhibition of Na_v_1.8 is mediated via an interaction between the toxin and DII’s voltage sensor [77,129]. A similar approach was used to study the interaction between Na_v_1.9 voltage sensors and scorpion or tarantula toxins. The voltage-sensor paddle motifs of Na_v_1.9 were transferred into the voltage-gated potassium channel K_v_2.1 [130]. This enabled examination of their interactions with tarantula and scorpion toxins [131]. The possibility that MrVIB could also interact with other domains cannot be excluded. Results from recent studies into MfVIA inhibition of Na_v_1.2 and other voltage-sensor toxins such as tarantula toxins may support the idea of multiple binding sites [132,133].

MfVIA, a novel μO-conotoxin, was recently identified from *Conus magnificus*. Similar to MrVIA and MrVIB, it is a hydrophobic peptide containing 32 residues, but has highest sequence homology to MrVIB [133]. MfVIA’s amino acid sequence is only slightly different to those of MrVIA and MrVIB. Surprisingly, this results in MfVIA having different potencies and selectivity towards mammalian VGSC subtypes from those of MrVIA and MrVIB. MfVIA is three-fold more potent towards Na_v_1.4 and five-fold less potent towards Na_v_1.2 than MrVIA or MrVIB. MfVIA inhibits TTX-R Na^+^ currents expressed in DRG neurons five times more strongly than those heterologously expressed in rNa_v_1.8. It also inhibits Na_v_1.4 and Na_v_1.8 at low nanomolar concentrations, whereas significantly higher toxin concentrations are needed to inhibit all other VGSC subtypes (Table 2) [133]. 

**Table 2:** Neuronal VGSC subtype selectivity and potency of μO-conotoxins.

μO-Conotoxins	*Conus* species	Number of residues	VGSC subtypes(IC_50_)	References
MrVIA	*C. marmoreus*	31	Na_v_1.7 (345 nM)	[123,134]
MrVIB*	*C. marmoreus*	31	Na_v_1.3 (1 μM),	[62,123,134]
			Na_v_1.7 (345 nM),	
			Na_v_1.8 (1–326 nM)	
MfVIA	*C. magnificus*	32	Na_v_1.3 (2175 nM),	[133]
			Na_v_1.7 (2317–5491 nM),	
			Na_v_1.8 (529 nM)	
LtVIIA	*C. litteratus*	29	N.D.	[135,136]
LtVIC	*C. litteratus*	28	N.D.	[135,^136^]

N.D., not determined. *commercial name CGX-1002 (Cognetix Inc.); entered pre-clinical trial for neuropathic pain (i.t./i.v.) [109].

The established model of μO-conotoxin mediated block of VGSC channels involves a single toxin molecule binding and inhibiting the channel. This is consistent with MfVIA inhibition of all VGSC subtypes except Na_v_1.2. The concentration–dependence for Na_v_1.2 inhibition was steeper than the expected Hill slope of −1 [133]. This may indicate that MfVIA interacts with more than one binding site. Another possibility might be that μO-conotoxins have slow on-rates that make it difficult to reach equilibrium at low peptide concentrations [133]. 

DNA sequencing of *C. litteratus* has revealed the existence of two novel μO-conotoxins, LtVIC and LtVIIA. These recombinant toxins inhibit Na^+^ currents in DRG neurons in a similar way to known μO-conotoxins. However, their subtype selectivity and structure–activity relationships are yet to be investigated [135,136,137]. 

MrVIA, MrVIB and MfVIA remain the only μO-conotoxins for which mammalian VGSC subtype selectivity data is available, albeit not across all VGSC subtypes. The unique selectivity profile of μO-conotoxins MfVIA, MrVIA and MrVIB [62,82,126,133] makes these peptides attractive leads for analgesic drugs [62,129]. Synthesis of μO-conotoxins does, however, pose significant challenges. This is mainly due to their highly hydrophobic nature and the difficulty of correctly forming their three disulfide bonds to the native isomer. While replacing cysteines with selenocysteines has helped with MrVIB folding [138], synthesis of the native molecule relies on a semi-selective approach [124,134], which produces a low overall yield. Nevertheless, a novel regioselective protocol has been developed for synthesizing the novel μO-conotoxin MfVIA that only uses a single HPLC purification step and increases peptide yields [133].

Another potential problem with μO-conotoxins is that their affinity for Na_v_1.4 and Na_v_1.8 is almost identical. This may limit their therapeutic effectiveness, unless derivatives of the toxins can make them more selective for Na_v_1.8 than Na_v_1.4. Stürzebecher *et al.* introduced a membrane-tethered isoform of MrVIA (t-MrVIA) into nociceptive neurones in mice, where it was successfully expressed on the cell surface [123]. TTX-R VGSC current densities, which were mainly Na_v_1.8-mediated, were reduced by 44 ± 7% in t-MrVIA transgenic mice, without up-regulation of TTX-S VGSC, VGCC or transient receptor potential channel expression in nociceptive neurones [123]. t-MrVIA transgenic mice had less inflammatory mechanical hypersensitivity and firing of cutaneous C-fibres sensitive to noxious cold temperatures, and were more insensitive to cold pain than wild-type mice [123]. Besides proving that MrVIA is analgesic, membrane-tethered MrVIAs could be used to study and manipulate VGSCs in specific cell types in the mammalian nervous system [123], further increasing the toxin’s selectivity.

## 4. β-Subunits Modulate the Effects of Conotoxins

Auxiliary β1-, β2-, β3- and β4-subunits, when individually co-expressed with Na_v_1.8 in *Xenopus* oocytes, increased the *k_on_* of μO-MrVIB inhibition of Na^+^ current by 3-, 32-, 2- and 7-fold, respectively [127]. β-subunits also modestly decreased the *k_off_* rate [127]. Different β-subunit expression rates, in combination with depolarizing prepulses, markedly accelerated MrVIB washout. Co-expression of β-subunits, in particular β2, with Na_v_1.8 α-subunits strongly influenced μO-conotoxin affinity for this subtype. However, expression of the a-subunit with auxiliary β-subunits did not appear to influence μ-conotoxin selectivity or affinity for Na_v_1.8 [107]. Whether or not this is also true for other members of this toxin family is yet to be determined [137].

## 5. Multiple Sites of μ- and μO-Conotoxin Action

Many animal toxins that target multiple pharmacologically distinct ion channels or receptors have been identified in recent years. For example, MrVIA and MrVIB have been reported to inhibit mollusc and mammalian VGSCs, as well as mollusc VGCCs [82,107]. However, Daly *et al.* showed that mammalian VGCCs in DRG neurons were unaffected by these toxins. Therefore, it can be assumed that the analgesic effects evoked by MrVIA and MrVIB are unrelated to VGCCs [125]. 

It was also recently shown that μ-conotoxin CnIIIC has multiple targets, including VGSCs and ligand-gated channels [98]. CnIIIC from *Conus consors* inhibited Na_v_1.4, with an IC_50_ of 1.3 nM, and α3β2 nicotinic acetylcholine receptor (nAChR) channels, with an IC_50_ of 450 nM. nAChR subtypes α7 and α4β2 were inhibited to a lesser extent, in the micromolar range [98]. The diversity of pharmacological interactions of conotoxins with different membrane receptors, transporters and ion channels should not be overlooked when developing new analgesics and other bioactive drugs.

## 6. Conotoxins—Analgesics of the Future?

Since their discovery, conotoxins have been of interest because of their high affinity and selectivity for VGSCs and other membrane receptors and ion channels. Neuropathic and inflammatory pain is associated with changes in VGSC activity and expression [25,139]. Because of this, several conopeptides are being investigated as anti-nociceptive drugs [73]. To date, however, no therapeutic drug that selectively targets a specific VGSC subtype is available. Despite the fact that several conotoxins have been and are currently being tested in clinical trials, only the w-conotoxin MVIIA from *Conus magus* has been approved as an analgesic drug (Ziconitide, Prialt^®^) to treat intractable pain [140,141,142].

μO-Conotoxins MrVIA, MrVIB and MfVIA block Na_v_1.8, which has a major role in pain, but does not appear to be important for other physiological functions [18]. As such, these peptides have potential as new analgesics and are the focus of several studies. In particular, MrVIB reduces pain without causing motor deficits in animal models [62]. Of the μ-conotoxins, SIIIA and KIIIA have both shown analgesic activity in mouse models [101,105]. However, both peptides also target skeletal muscle sodium channels, which reduces their analgesic potential in their native form. Nonetheless, specifically designed derivatives of these peptides have the potential to increase selectivity towards VGSC pain targets [100].

Side effects from the native toxin must be expected and should be carefully assayed in detail. For example, μO-conotoxins also affect the muscle VGSC subtype Na_v_1.4, which may limit their therapeutic effectiveness [62,128]. Side effects are also a particular consideration for μ-conotoxins, because they target muscle and neuronal VGSC subtypes [99]. However, a major hurdle in developing conotoxins as analgesic drugs is creating a drug that targets a single VGSC subtype. So far, no toxin selective for only one VGSC subtype has been discovered. Furthermore, pharmacological interactions of conotoxins with membrane receptors and ion channels other than their target (e.g., VGSCs) can be underestimated. The same is true for other animal peptide toxins, for example spider toxins [143,144,145]. 

Other problems associated with conotoxins include their challenging chemical synthesis and folding, which make it difficult to produce conopeptides in large quantities. Orally active drugs are also difficult to produce, although they are most desirable, because they would be better accepted by patients and doctors [146]. For example, MVIIA (Ziconitide, Prialt^®^) requires intrathecal administration which limits its range of applications. Despite these complications, conotoxins that inhibit VGSCs and have some subtype selectivity are valuable tools for studying ion channels and their physiological role. This will contribute to the development of novel drugs to improve pain management and treat neurological disorders.
